# Analysis of the positive results and influencing factors of hepatitis B antibody in hospitalized neonates with AgHBs positive mothers

**DOI:** 10.3389/fped.2022.1042435

**Published:** 2022-12-22

**Authors:** Yu xiao Min, Ying Gao, Chun tian Liu, Xiao yu Lu, Xiao chun Chen

**Affiliations:** Department of Neonatology, The Second Affiliated Hospital and Yuying Children's Hospital of Wenzhou Medical University, Wenzhou, China

**Keywords:** hepatitis b virus 乙型肝炎病毒 hepatitis b virus hepatitis b virus, newborn 新生儿 newborn, immune effect 免疫效应 immune effect, influencing factors 影响因素 influencing factors, hepatitis b vaccine 乙型肝炎疫苗 hepatitis b vaccine hepatitis b vaccine

## Abstract

**Purpose:**

To investigate the results of positive antibody to hepatitis surface antigen(anti-HBs)in hospitalized neonates whose mothers were hepatitis B surface antigen (AgHBs) positive and to explore the influencing factors.

**Method:**

The study subjects were hospitalized neonates whose mothers were positive for AgHBs. According to the serological test results of five immune markers of hepatitis B virus (HBV), they were divided into positive for anti-HBs and negative for anti-HBs. Retrospective analysis of relevant factors affecting results of anti-HBs.

**Result:**

269 cases (80.78%) were positive for anti-HBs and 64 cases (19.22%) were negative for anti-HBs. Univariate analysis results: the number of hepatitis B immunoglobulin (HBIG) injections after birth, whether HBIG was injected within 6 h, whether Hepatitis B vaccine (Hep B) was injected within 6 h, whether combined immunization within 12 h, whether Hep B was vaccinated on time after discharge, whether preterm birth, and whether low birth weight infants were statistically significant (*P* < 0.05). The results of binary logistic regression analysis: HBIG injection time ≤6 h (OR = 0.213), combined immunization time ≤12 h (OR = 0.024) were protective factors; premature infants (OR = 7.175), ALB/GLO (OR = 9.792) and failure to complete three vaccinations on time (OR = 12.659) were risk factors (*P* < 0.05).

**Conclusion:**

Although China has implemented a national immunization program, vaccination of hospitalized neonates whose mothers are positive for AgHBs has not been effective. Therefore, it is recommended to strengthen training for medical staff and families to ensure that neonates can complete the three doses of vaccination on time after discharge from the hospital and to strengthen follow-up for premature infants.

## Introduction

There are about 257 million people in the world with chronic hepatitis B (CHB) infection, accounting for about 3.5% of the total population (1), China is a high incidence area of CHB infection, with about 90 million CHB infected people. Studies have shown that 50% of hepatitis B virus (HBV) infections come from mother-to-child transmission (2). 90% of neonates infected with HBV develop chronic infection. Comprehensive and high-quality implementation of hepatitis B vaccine (Hep B) vaccination is an effective means to reduce mother-to-child transmission of hepatitis B in neonates (3). In 2002, China has popularized 3 doses of Hep B vaccination, which are: 0 (within 24 h after birth), 1 month, and 6 months. Neonates whose mothers were hepatitis B surface antigen (AgHBs) positive were routinely injected with a dose of 100 IU of hepatitis B immunoglobulin (HBIG) before the first dose of vaccine (4). However, studies have reported that 5 to 20% of neonates fail or fail to respond to vaccination (5). Newborns who need to be admitted to a neonatal intensive care unit (NICU) due to critical conditions such as preterm birth, neonatal asphyxia, and low birth weight cannot be vaccinated on time during hospitalization, and their families lack knowledge about Hep B vaccination. The rate of immune response failure or non-response after vaccination is significantly higher than that of non-hospitalized neonates (6). How to take effective intervention measures for hospitalized neonates, implement Hep B vaccination, and improve the immune response ability after vaccination is an important way to cut off mother-to-child transmission.

Therefore, this study took hospitalized neonates whose mothers were AgHBs positive as the research object, and analyzed the factors affecting the positive results of anti-HBs. Provide a basis for further development of interventions.

## Objects and methods

### Object of investigation

This study was a retrospective study. Convenience sampling was used to select neonates born in the obstetrics department of a tertiary hospital in Wenzhou City from January 2018 to April 2021 and admitted to the NICU as the survey subjects. Inclusion criteria: ① hospitalized neonates with AgHBs positive mothers; ② Directly admitted to the neonatal intensive care unit (NICU) after birth; ③ First dose of HBIG and Hep B completed in the NICU. Exclusion criteria: ① The mother suffers from other serious diseases, infectious diseases; ② neonatal death.

### Grouping basis

According to the serological results of the five immunological markers of hepatitis B virus (referred to as serological test results), they were divided into two groups: positive for anti-HBs and negative for anti-HBs.

### Survey methods

Search CNKI, Wanfang database, VIP database, PubMed and other databases to determine the relevant factors affecting the expression of antibodies after hepatitis B vaccination, screen variables based on clinical and expert opinions, and formulate general information questionnaires. ① Demographic data: neonatal gender, gestational age, birth weight, mode of delivery, mother's and father's educational level, feeding method, mother's history of abortion, and parent's disease history. ② HBIG injection: The number of neonates who received HBIG injection once or ≥2 times after birth and the number of neonates who completed the first injection within 6 h. ③ Hep B vaccination: the number of neonates who completed injection of Hep B within 6 h. ④ Number of neonates with combined immunization within 12 h (The first dose of Hep B was administered within 12 h after HBIG injection). ⑤ Blood biochemical examination: blood biochemical examination before injection of HBIG and Hep B (Measured immediately after birth), serological test results after completing three injections of Hep B as planned. ⑥ Whether Hep B vaccination is on time after discharge (1, 6 months old) (7), and the institution that inject hepatitis B vaccine.

### Data collection and quality control methods

Data were collected by two uniformly trained master's degree students in nursing through electronic medical records and entered into an Excel sheet after double-checking.

### Statistical methods

Statistical analysis was performed using SPSS 26.0 software. The Kolmogorov-Smirnov test and the normal distribution map were used to verify whether the continuous variables conformed to the normal distribution, and the data with normal distribution were described by the mean ± standard deviation. Non-normally distributed measurement data were expressed by the median and interquartile range, and the comparison between the two groups was performed by two independent samples rank sum test. Enumeration data were expressed by frequency, percentage or percentage, and the comparison between groups was performed by chi-square test. Serological test was used as the dependent variable, and binary logistic regression was performed to explore the influencing factors of serological test results. *P* < 0.05 was considered to be statistically significant.

## Results

### Status of general information and serum test results of the survey respondents

A total of 506 neonates were included in this study, and 48 were lost to follow-up. Among them, 3 patients died, 9 patients had incomplete case data, and 36 patients had incomplete information. Of the 458 neonates who were finally included, 125 (27.29%) were not tested for serology, and 333 (72.70%) were tested for hepatitis B antibodies. Among the newborns tested for serum antibodies, 269 (80.78%) were antibody positive and 64 (19.22%) were antibody negative.

### Analysis of factors influencing anti-HBs in hospitalized neonates

#### Univariate analysis of anti-HBs in hospitalized neonates

The univariate analysis results of HBV surface antibody positive and negative groups showed: the number of HBIG injections after birth, whether HBIG was injected within 6 h, whether Hep B was injected within 6 h, whether combined immunization within 12 h, whether Hep B was vaccinated on time after discharge, whether it was full-term, whether it was low Compared with the birth weight infants, the difference was statistically significant (*P* < 0.05), as shown in Table 1. The blood biochemistry showed that the ratio of AS/AL, alkaline phosphatase, globulin and white globulin was statistically significant (*P* < 0.05), as shown in Table 2.

**Table 1:** Univariate analysis of anti-HBs in hospitalized neonates.

item [example (percentage, %)].	Negative for anti-HBs (*n* = 64)	Positive for anti-HBs (*n* = 269)	Test statistics	*P* value
Gender			0.289	0.591
Male	39 (60.90)	154 (57.20)		
Female	25 (39.10)	115 (42.80)		
Number of HBIG injections after birth			3.871	0.049
1	54 (84.40)	195 (72.50)		
2	10 (15.60)	74 (27.50)		
HBIG was injected within 6 h	47 (73.40)	247 (91.80)	16.898	0.000
HepB was injected within 6 h	6 (9.40)	195 (72.50)	86.076	0.000
combined immunization within 12 h	4 (6.20)	215 (79.90)	124.645	0.000
HepB was vaccinated on time after discharge	11 (17.20)	206 (76.60)	80.338	0.000
premature birth.	61 (95.30)	208 (77.30)	10.776	0.001
low birth weight infants	57 (89.10)	176 (65.40)	13.745	0.000
Parents’ hepatitis B status			0.462	0.497
mother hepatitis B	51 (79.70)	224 (83.30)		
Both parents have hepatitis B	13 (20.30)	45 (16.70)		
Mother's educational level			2.273	0.321
Bachelor degree or above	17 (26.60)	56 (20.80)		
Senior high school and junior college	26 (40.60)	98 (36.40)		
Junior high school and below	21 (32.80)	115 (42.80)		
Father's educational level			0.384	0.825
Bachelor degree or above	10 (15.60)	51 (19.00)		
Senior high school and junior college	26 (40.60)	105 (39.00)		
Junior high school and below	28 (43.80)	113 (42.00)		
breastfeeding	33 (51.60)	138 (51.30)	0.001	0.970
Mode of delivery			0.259	0.611
cesarean section	32 (50.50)	144 (53.50)		
natural delivery	32 (50.50)	125 (46.50)		
History of abortion	14 (21.90)	67 (24.90)	0.258	0.611
Vaccination site			0.393	0.822
epidemic prevention station	11 (17.20)	54 (20.10)		
Community	38 (59.40)	149 (55.40)		
hospital	15 (23.40)	66 (24.50)		

**Table 2:** Univariate analysis of anti-HBs blood biochemistry in hospitalized neonates.

Parameters [M (P25, P75)]	Negative for anti-HBs (*n* = 64)	Positive for anti-HBs (*n* = 269)	Test statistics	*P* value
ALT (U/L)	7 (4,9)	7 (5,10)	−0.606	0.544
AST (U/L)	43.5 (33,65.75)	43 (30,59)	−1.097	0.272
AS/AL	6.8 (5,9.475)	5.75 (4.4,8.345)	−2.247	0.025
ALP (U/L)	186.5 (164.25,243)	223 (179,279)	−2.389	0.017
GGT (U/L)	171.5 (124.5,288)	196 (130.5,315)	−0.940	0.347
TP (g/L)	45.95 (43.5,51.85)	45.5 (41.9,49.7)	−1.377	0.168
ALB (g/L)	29.9 (27.925,32.75)	30 (27.9,32.4)	−0.413	0.679
GLO (g/L)	16.3 (14.7,19.275)	15.3 (13.25,17.4)	−3.085	0.002
ALB/GLO	1.8 (1.7,2)	2 (1.8,2.15)	−4.712	0.000
TBIL (umol/L)	59.85 (37.625,87.15)	58.3 (42.35,81.45)	−0.121	0.903
DBIL (umol/L)	8.3 (6.7,10.875)	8.3 (6.7,10.4)	−0.100	0.920
IBIL (umol/L)	59.05 (32.125,80.4)	51.6 (32.5,72.05)	−0.883	0.377
BUN (mmol/L)	3.81 (3,5.525)	4 (3.175,5.5)	−0.358	0.721
CREA (umol/L)	62.25 (55.1,77.4)	61.7 (49.4,72.55)	−0.891	0.373
BU/CR	0.06 (0.05,0.09)	0.07 (0.0585,0.08)	−0.798	0.425
UA (umol/L)	341.5 (255.5,390.5)	357 (268,451.5)	−1.055	0.292
CK (U/L)	216.5 (145,434)	204 (121.5,348.5)	−1.232	0.218
LDH (U/L)	536.5 (433.25,655.75)	512 (407,640)	−0.873	0.383
CA (mmol/L)	2.065 (1.86,2.2375)	2.12 (1.91,2.28)	−1.284	0.199
IP (mmol/L)	1.825 (1.6125,2.1)	1.87 (1.64,2.07)	−0.652	0.514
MG (mmol/L)	0.815 (0.7525,0.9075)	0.81 (0.74,0.905)	−0.782	0.434
NA (mmol/L)	137.85 (136.025,140)	137.3 (135.6,139.5)	−1.127	0.260
K (mmol/L)	4.705 (4.3125,5.2475)	4.73 (4.32,5.16)	−0.144	0.886
CL (mmol/L)	106.4 (103.6,109.175)	106.9 (104.35,109.1)	−0.815	0.415

Abbreviations: ALT(U/L), Alanine transaminase (7∼40); AST (U/L), Aspartate transaminase (13∼35); AS/AL, Alanine transaminase/Aspartate transaminase (/); ALP (U/L), Alkaline phosphatase (<1107); GGT (U/L), Glutamyl transpeptidase (7∼45); TP(g/L), Total protein (65∼85); ALB (g/L), Albumin (40∼55);GLO(g/L), Globulin (20∼40); ALB/GLO, Albumin/Globulin (1.2∼2.4); TBIL (umol/L), Total bilirubin (5∼21); DBIL (umol/L), Direct bilirubin (1.7∼6.8); IBIL (umol/L), Indirect Bilirubin (< 17.3); BUN (mmol/L), Blood Urea Nitrogen (3.1∼8); CREA (umol/L), Creatinine (41∼73); BU/CR, Blood Urea Nitrogen/Creatinine (0.03∼0.15); UA (umol/L), Uric acid (155∼357); CK (U/L), Creatine kinase (26∼140); LDH (U/L), Lactate dehydrogenase (120∼250); CA (mmol/L), Calcium (2.23∼2.8); IP (mmol/L), Phosphorus (1.45∼2.1); MG (mmol/L), Magnesium (0.75∼1.02); NA (mmol/L), Sodium (137∼147); K (mmol/L), Potassium (3.5∼5.3); CL (mmol/L), Chlorine (99∼110).

#### Binary logistic regression analysis of anti-HBs in hospitalized neonates

Taking hepatitis B antibody test results as the dependent variable, the independent variables with statistical significance (*P* < 0.05) in the univariate analysis were included in the binary logistic regression analysis. The results showed that whether HBIG was injected within 6 h, whether combined immunization within 12 h, whether the vaccine was vaccinated on time after discharge, whether it was full-term, and the white blood cell ratio were the independent influencing factors of hepatitis B antibody production in hospitalized neonates, as shown in Table 3.

**Table 3:** Binary logistic regression analysis of anti-HBs in hospitalized neonates.

independent variable	*B*	standard error	Walds *χ*2 value	*P* value	OR value	95% confidence interval
Constant	−0.867	2.580	0.113	0.737	0.420	
whether HBIG was injected within 6 h	−1.545	0.654	5.577	0.018	0.213	0.059∼0.769
whether combined immunization within 12 h	−3.725	0.617	36.430	0.000	0.024	0.007∼0.081
Whether to vaccinate Hep B on time after discharge	2.538	0.475	28.588	0.000	12.659	4.992∼32.100
premature birth	1.971	0.799	6.082	0.014	7.175	1.498∼34.351
A/G	2.282	0.981	5.408	0.020	9.792	1.431∼66.990

Note: Omnibus test of this model χ2 = 7.452, *P* < 0.001; Hosmer Lemeshow test χ2 = 3.810, *P* = 0.577; R2 = 0.690.

#### The relationship between blood biochemical test results and neonatal anti-HBs expression

Although there is no significant difference between negative and positive for anti-HBs groups in these indicators (AST, ALT, and ALB) alone, their ratios are significantly different (Table 3).

**Figure 1:**
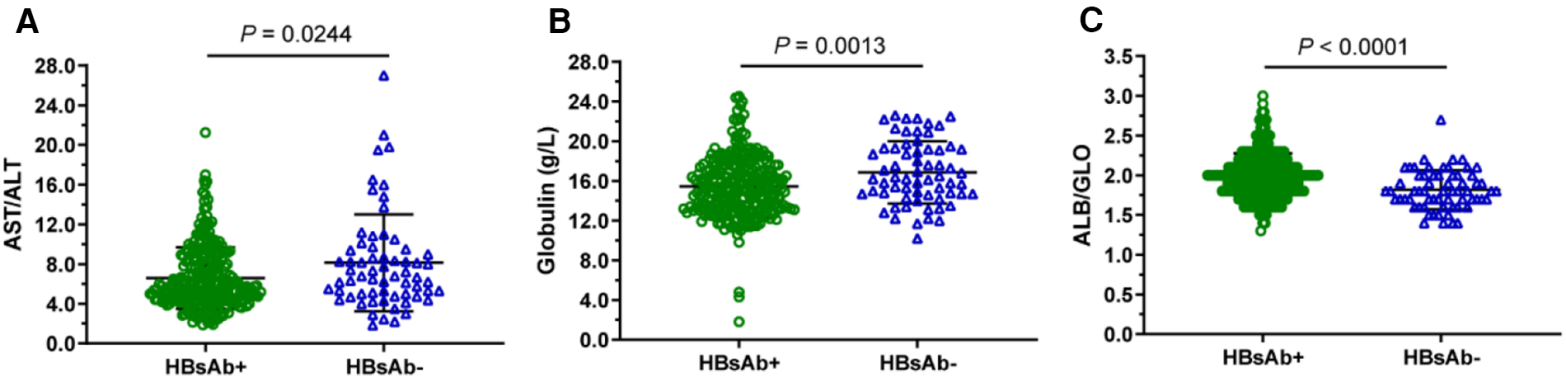
Difference of serum AST/ALT, globulin, or ALB/Glo levels between two groups: positive for anti-HBs and negative for anti-HBs.

Difference of serum AST/ALT, Globulin, or ALB/ Glo levels between two groups: positive for anti-HBs and negative for anti-HBs. (Figure 1). Serum AST/ALT level is significantly lower in positive for anti-HBs subjects compared with that in negative group subjects (Figure 1A); similarly, Globulin level significantly lower in positive group subjects (Figure 1B). However, the ALB/Glo is significantly higher in positive for anti-HBs subjects compared with that in negative for anti-HBs subjects (Figure 1C). Data are expressed as scatter plots, in which the middle of the black solid line is the median, and the up and down horizontal lines represent the 25th and 75th percentiles, respectively.

**Figure 2:**
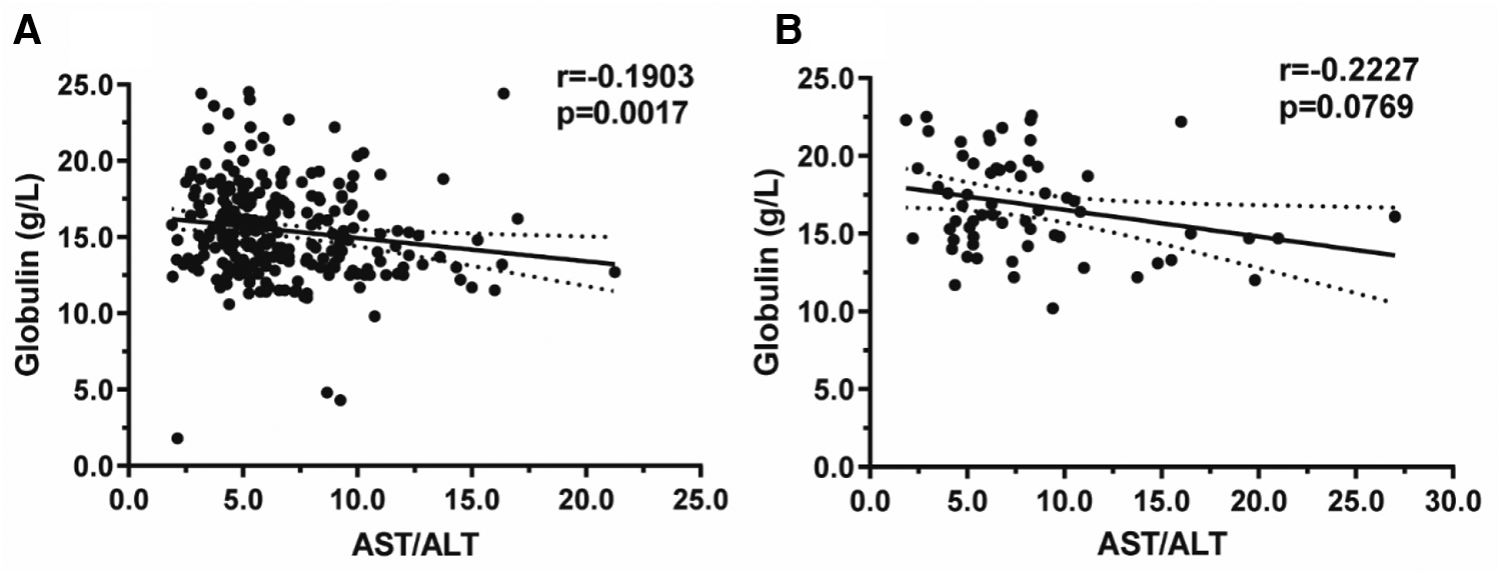
Relationship between AST/ALT and globulin levels between two groups: positive for anti-HBs and negative for anti-HBs.

The relationship between blood biochemical AST/ALT and GLO levels in 333 neonates was analyzed using Spearman's correlation analysis, and there was a statistically negative correlation in the positive group (*r*^2 ^= −0.1903, *p *= 0.0017; Figure 2A), but in negative group, there was no significant correlation between AST/ALT and Glo levels (*r*^2 ^= 0.0132, *p *= 0.3039; Figure 2B).

## Discussion

### Vaccination failure rate of hospitalized neonates is high, mainly preterm infants

The results of this study showed that 64 (19.22%) neonate were negative for anti-HBs, of which 61 were premature. Peng et al. (8) found that premature delivery was a risk factor for HBV infection through a retrospective cohort study. Preterm infants are 1.36 times more unresponsive to Hep B than term infants (9). This is related to the physiological characteristics of premature infants, the immature immune system, the total number of peripheral blood T lymphocytes and the proficiency of each subgroup are low (10). To improve the success rate of the Hep B vaccination response, the United States (11) and the United Kingdom (12) recommend a four-dose hepatitis B vaccination schedule for preterm infants with birth weight <2,000 g [0 (within 12 h after birth), 1, 2, and 12 months old]. China's 2021 version of “Immunization Procedures and Instructions” (13) clearly stated that “for newborns born to AgHBs positive mothers weighing less than 2000g, the first dose of Hep B should be vaccinated as soon as possible (within 12 h) after birth. When the corrected gestational age of the newborn reaches 1 month, 2 months, and 7 months of age, 3 doses of Hep B vaccination should be completed according to the procedure.”.

### HBIG injection within 6 h is a protective factor for anti-HBs positive

The results of this study showed that postnatal HBIG injection time ≤6 h was a protective factor for the production of anti-HBs in hospitalized neonates. According to existing guidelines (14): HIBG needs to be used within 12 h after birth (in theory, the earlier the better). Its active ingredient is anti-HBs, which starts to work 15–30 min after intramuscular injection, and the protective anti-HBs can be maintained for at least 42–63 days. Zheng Hui et al. (15) established a scenario model for the existing domestic hepatitis B prevention intervention policies in China, and the prediction results found that if HBIG was given to newborns of AgHBs positive mothers according to the current prevention policy, antiviral treatment (Tenovir) to hepatitis B-positive pregnant women, China will be able to eliminate mother-to-child transmission intervention strategies by 2030. Hongyu Huang et al. (16) conducted an observational study of 1,140 infants born to HBV-infected mothers. The results showed that although the success of immune prophylaxis is closely related to the level of HBV DNA in the mother, the early neonatal period (within 1 h after birth) the use of HIBG may provide better protection against mother-to-child transmission of hepatitis B.

### Combined immunization within 12 h is a protective factor for anti-HBs positive

The results of this study showed that the time interval between the injection of Hep B and HBIG ≤12 h was a protective factor for the production of anti-HBs. This is consistent with the research results of China's 2016–2017 National Project on Prevention of Mother-to-Child Hepatitis B Transmission. Children who received combined immunization within 12 h to 24 h after birth were 2.9 times more likely to be infected with HBV than newborns vaccinated within 12 h (17). The blocking effect of combined immunization is better than that of Hep B alone, and the protection rate of combined immunization within 1 h of birth can reach 97% (16, 18), A prospective, multi-center observational study of neonates born to AgHBs positive mothers found that the mother-to-child transmission rate was 0.9% when the neonates were injected with HBIG and Hep B within an average of 0.17 h after birth (16). The guidelines recommend (7) that neonates must inject HBIG intramuscularly within 12 h after birth, and at the same time inject the first dose of Hep B intramuscularly in different parts (the sooner the better, the most within minutes). At present, most neonates can be vaccinated with Hep B within 24 h after birth, and few can be vaccinated within 1 h. It is recommended to optimize the workflow of combined immunization to ensure that newborns are vaccinated within 1 h after birth.

### Failure to vaccinate Hep B on time after discharge is a risk factor for anti-HBs positive

The results of this study showed that the failure to receive Hep B on time after discharge was a risk factor for the development of anti-HBs. 216 (65.17%) neonates received Hep B on time after discharge. Timely vaccination can effectively reduce the risk of HBV infection in children. After the first dose of vaccine, most newborns remain negative for anti-HBs or below the lower limit of detection, which protects the organism from HBV infection for 35 to 40 days; The infant developed an immune response after receiving the second dose of the Hep B vaccine, and the anti-HBs was positive. A third dose of the vaccine extends protection to more than 30 years (19, 20). In the survey, it was found that the main reasons for the delay in vaccination were neonatal colds, the containment of the COVID-19 epidemic, and fear of vaccine side effects. Studies have shown that Hep B vaccine has no effect on neonatal cardiac electrical activity and cerebral blood flow (21), so if there is no special reason, it is recommended that neonates be vaccinated with Hep B within a specified time after discharge. Based on this phenomenon, we are strengthening the awareness of community workers about the timely vaccination of Hep B and increasing the rate of timely vaccination of newborns in the jurisdiction.

### Preterm birth is a risk factor for anti-HBs positive

The results of this study show that preterm birth is a risk factor for the development of anti-HBs. There were 269 (80.78%) neonates with anti-HBs positive in this study, of which 61 (22.70%) were term infants and 208 (77.30%) were premature infants. A retrospective cohort study showed that pregnant women who were positive for both AgHBs and AgHBc had a 47% increased overall risk of preterm delivery compared with uninfected pregnant women (22), which may explain the preponderance of preterm births in our present study. Although studies have shown that the immunity of preterm infants eventually converges to that of term infants (23), the proportion of full-term infants producing anti-HBs is higher than that of preterm infants. Freitas’s (24) findings are consistent with ours, Term infants have a relatively well-developed immune system, and can obtain maternal immune globulin transferred via the placenta in the third trimester of pregnancy, and the B cell differentiation pathway is more mature (25), which makes term infants have a better response to Hep B.

### The relationship between blood test results and neonatal hepatitis B antibody production results

**Figure 3:**
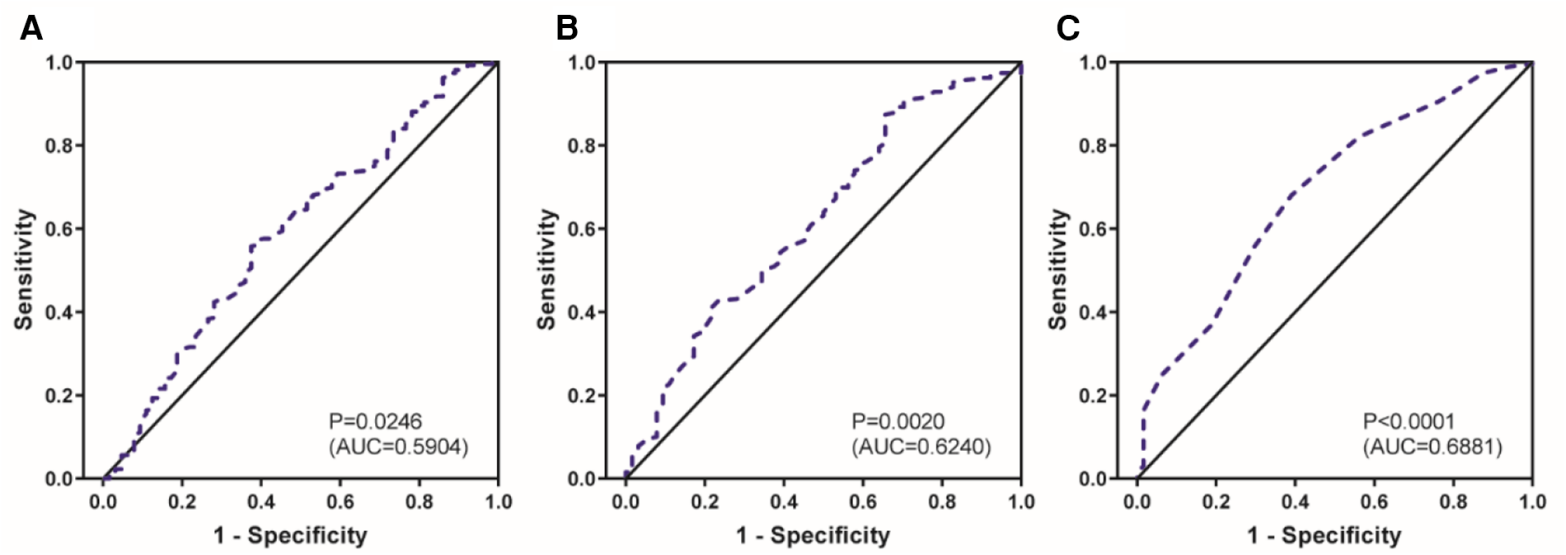
Receiver operating characteristic prediction analysis chart.

The relationship between anti-albumin antibodies and AgHBe in hepatitis B patients was previously studied by M Bozic et al. (26), and the experimental results revealed that the AgHBe positivity rate was 60.5% in sera containing anti-albumin antibodies. Similar to the results of this study (Figure 1) which showed that ALB/GLO may have a positive correlation with the production of anti-HBs in neonates, the level of ALB/GLO in positive for anti-HBs preterm infants was significantly higher than in negative for anti-HBs preterm infants as shown in Figure 1(C), the findings support that albumin plays an important role in the viral life cycle. Meanwhile, Figure 1 shows that AST/ALT in positive for anti-HBs preterm infants is lower than that in the negative for anti-HBs group. However, there is no relevant literature report so far. The sample size of this study is limited, and it depends on more basic experimental research. Figure 2 further shows that the increase in AST/ALT in the positive for anti-HBs neonatal group was negatively correlated with the increase in GLO. We further performed Roc analysis to assess the ability of AST/ALT, GLO, and ALB/GLO levels in neonates to predict hepatitis B antibody production. As shown in Figure 3 GLO (AUC = 0.6240) ALB/GLO (AUC = 0.6881) had consistently higher AUC values. It has been shown (27) that the globulin-platelet model can predict liver fibrosis or the development of cirrhosis in patients with hepatitis B. Therefore, this study provides a better idea of the prediction model of serum AST/ALT and GLO for newborns with maternal AgHBs which can be tested and applied in clinical practice in the future on an evidence-based basis.

## Conclusion

Overall, because China is a big country with hepatitis B, the clinical demand for HBIG is very large. Although time requirements are written in the immunization program, few family members of hospitalized neonates are able to perform hepatitis B vaccination as required. The results of this study provide new insights and possibilities for drug development, intervention and treatment strategies for HBV. It is expected that clinical workers will strengthen the immunization process and management in order to further improve the effectiveness of Hep B vaccination for hospitalized newborns and to interrupt mother-to-child transmission at an early stage.

## Limitation

This study was limited to a single-center study in one tertiary hospital in one city, and the study population was mainly preterm infants, without grouping studies for different gestational ages. It is recommended that future multicenter and large-sample studies be conducted to further analyze the variability of preterm infants at different gestational ages.

## Data Availability

The original contributions presented in the study are included in the article/Supplementary Material, further inquiries can be directed to the corresponding author/s.
